# 20. Risk Factors for Breakthrough Cytomegalovirus (CMV) Infection and *De Novo* Resistance in Hematopoietic Cell Transplantation (HCT) Recipients Receiving Letermovir Prophylaxis

**DOI:** 10.1093/ofid/ofab466.020

**Published:** 2021-12-04

**Authors:** Danniel Zamora, Garrett Perchetti, Melinda Biernacki, Hu Xie, Jared L Castor, Laurel Joncas-schronce, Rachel Blazevic, Wendy Leisenring, Meei-Li Huang, Keith Jerome, Paul J Martin, Michael Boeckh, Alexander L Greninger

**Affiliations:** 1 University of Washington, Seattle, Washington; 2 Fred Hutch, Seattle, Washington; 3 Fred Hutchinson Cancer Research Center; University of Washington, Seattle, Washington; 4 Fred Hutchinson Cancer Research Center, Seattle, WA

## Abstract

**Background:**

Subclinical CMV reactivation on letermovir prophylaxis may be important for CMV-specific immune reconstitution after HCT (Zamora et al. *Blood* 2021) but concerns remain regarding the development of antiviral resistance. Here we analyze risk factors associated with breakthrough CMV infection on letermovir and describe the incidence of *de novo* letermovir resistance.

**Methods:**

All CMV-seropositive, allogeneic HCT recipients who received letermovir prophylaxis from 10/2018-2020 were analyzed. Weekly proportions and cumulative incidences of CMV reactivation in the first 100 days post-HCT were calculated at different levels. Clinically significant CMV infection was treated preemptively with (val)ganciclovir or foscarnet. Univariable/multivariable Cox regression models for breakthrough CMV reactivation at each viral threshold were performed. Patients with CMV reactivation ≥ 200 IU/mL were tested by UL56 sequencing to identify *de novo* letermovir resistance.

**Results:**

Two hundred thirty HCT recipients who received letermovir prophylaxis were identified. Weekly proportions and cumulative incidences of CMV reactivation are shown in **Figure 1**. Nine of 15 patients with CMV reactivation had sufficient serum for letermovir resistance testing. One C325Y mutation was identified in an umbilical cord blood transplant recipient who developed 4 weeks of CMV DNAemia with a peak of 2512 IU/mL. The patient received 56 days of letermovir prior to reactivation and responded to treatment initially with foscarnet (due to cytopenias) followed by ganciclovir. Greater cumulative steroid exposure was associated with increased risk of CMV reactivation and the association remained statistically significant at any level (adjusted Hazard Ratio [aHR] 10.8 mg/kg*days, 95% confidence interval [CI] 5.18-22.7) and ≥ 150 IU/mL (aHR 15.9 mg/kg*days, 95% CI 7.07-35.6) after adjusting for underlying disease and GVHD prophylaxis (**Figure 2**).

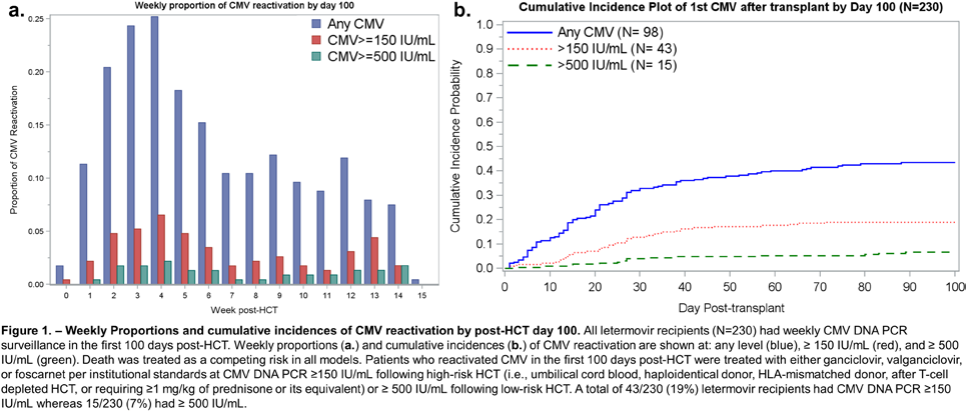

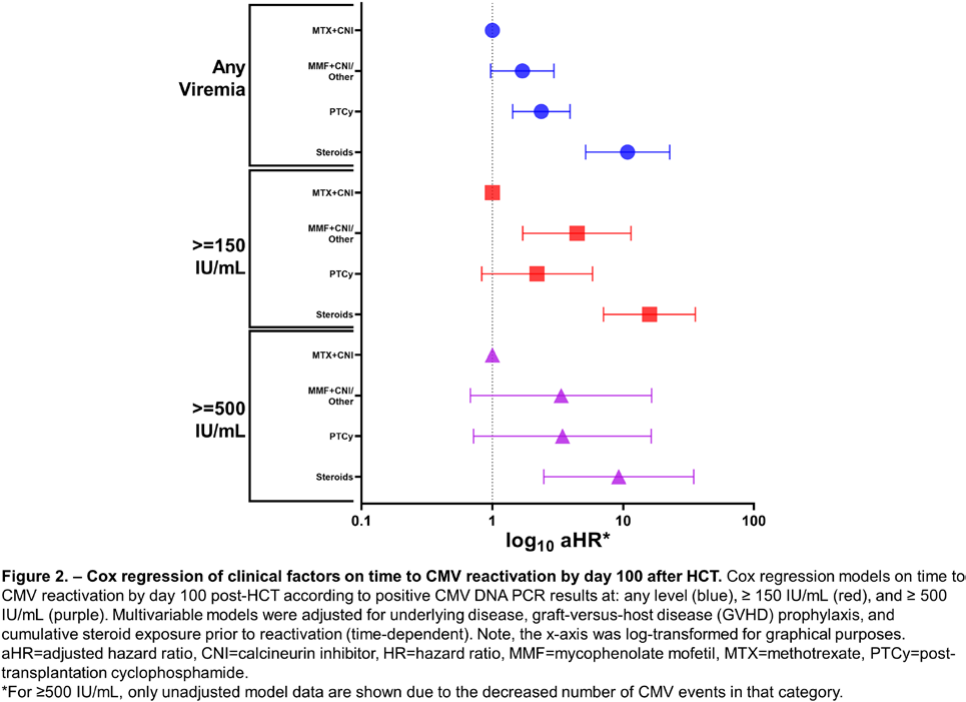

**Conclusion:**

Letermovir prophylaxis was effective at preventing clinically significant CMV infection but subclinical reactivation continued to occur. Cumulative steroid exposure was the strongest risk factor for reactivation while on letermovir. Development of *de novo* letermovir resistance on prophylaxis occurred infrequently.

**Disclosures:**

**Michael Boeckh, MD PhD**, **AlloVir** (Consultant)**Ansun Biopharma** (Grant/Research Support)**Astellas** (Grant/Research Support)**EvrysBio** (Consultant, Other Financial or Material Support, Options to acquire equity, but have not exercised them)**Gilead Sciences** (Consultant, Grant/Research Support)**GlaxoSmithKline** (Consultant)**Helocyte** (Consultant, Other Financial or Material Support, Options to acquire equity, but have not exercised them)**Janssen** (Grant/Research Support)**Kyorin** (Consultant)**Merck** (Consultant, Grant/Research Support)**Moderna** (Consultant)**Symbio** (Consultant)**Takeda (formerly known as Shire**) (Consultant, Grant/Research Support)**VirBio** (Consultant, Grant/Research Support) **Alexander L. Greninger, MD, PhD**, **Abbott** (Grant/Research Support)**Gilead** (Grant/Research Support)**Merck** (Grant/Research Support)

